# Identification of Suitable Internal Control miRNAs in Bovine Milk Small Extracellular Vesicles for Normalization in Quantitative Real-Time Polymerase Chain Reaction

**DOI:** 10.3390/membranes13020185

**Published:** 2023-02-02

**Authors:** Md. Matiur Rahman, Ryoka Nakanishi, Fumi Tsukada, Shigeo Takashima, Yoshiko Wakihara, Yuji O. Kamatari, Kaori Shimizu, Ayaka Okada, Yasuo Inoshima

**Affiliations:** 1 Laboratory of Food and Environmental Hygiene, Gifu University, Gifu 501-1193, Japan; 2 Department of Medicine, Faculty of Veterinary, Animal and Biomedical Sciences, Sylhet Agricultural University, Sylhet 3100, Bangladesh; 3 Division of Genomics Research, Life Science Research Center, Gifu University, Gifu 501-1193, Japan; 4 Institute for Glyco-Core Research (iGCORE), Gifu University, Gifu 501-1193, Japan; 5 The United Graduate School of Drug Discovery and Medical Information Sciences, Gifu University, Gifu 501-1193, Japan; 6 Division of Instrumental Analysis, Life Science Research Center, Gifu University, Gifu 501-1193, Japan; 7 Education and Research Center for Food Animal Health, Gifu University (GeFAH), Gifu 501-1193, Japan; 8 Joint Graduate School of Veterinary Sciences, Gifu University, Gifu 501-1193, Japan

**Keywords:** bovine milk small extracellular vesicles, internal control microRNA, normalization in quantitative polymerase chain reaction, RNA extraction kit

## Abstract

This study aimed to identify a suitable RNA extraction kit and stable internal control microRNA (miRNA) in bovine milk small extracellular vesicles (sEVs) for a quantitative polymerase chain reaction (qPCR) analysis. Two RNA extraction kits, miRNeasy Micro Kit, and Maxwell RSC miRNA Tissue Kit, were compared and evaluated using bovine milk sEVs via qPCR analysis. Five miRNAs, bta-miR-29a, bta-miR-200a, bta-miR-26b, hsa-miR-27b-3p, and hsa-miR-30b-5p, were selected by microarray analyses, and their cycle threshold (Ct) values were further evaluated mathematically using geNorm, NormFinder, BestKeeper, and ∆Ct algorithms. The results revealed that both the miRNeasy Micro Kit and Maxwell RSC miRNA Tissue Kit are useful for the efficient recovery of RNA from bovine milk sEVs. According to the final stability ranking analyzed by RefFinder, hsa-miR-27b-3p and bta-miR-29a can be used as suitable internal control miRNAs in bovine milk sEVs. The study also indicated that using a suitable internal control miRNA may improve the reliability and accuracy of the qPCR analysis for normalization in bovine milk sEVs. To the best of our knowledge, this is the first study to uncover the suitable internal control miRNAs in bovine milk sEVs.

## 1. Introduction

Small extracellular vesicles (sEVs) are membranous nanoparticles that are found in all bodily fluids, such as blood, urine, milk, etc. [1] sEVs contain microRNAs (miRNAs), mRNAs, DNAs, proteins, and lipids; therefore, they mediate cell-to-cell communication by fusion with the target cells and the transfer of cargo RNA encapsulated in bovine milk sEVs [2]. miRNAs are short endogenous non-coding RNAs that are widely expressed in the genomes of animals [3]. Previous studies have reported that alterations in miRNA expression are useful for understanding the pathophysiological status of hosts [4,5]. For example, hsa-miR-424-5p in bovine milk sEVs are useful as biomarkers for the identification of cattle with a high risk of bovine leukemia virus (BLV) transmission and enzootic bovine leukosis (EBL), respectively [6,7]. Thus, miRNAs play important roles in defining both normal and pathologically specific gene expression patterns. RNA extraction is a crucial step because it can affect detection assays and experimental techniques, as well as the quality of results and data robustness [8]. Currently, a number of miRNA extraction kits are available in the market, which are generally designed for biofluids such as plasma, serum, or cells [9,10]. However, the commercial kits available for miRNA extraction from bovine milk sEVs are limited. Previously, we identified miRNA biomarkers in milk sEVs for cattle with a high risk of BLV transmission and EBL cattle [6,7]. However, there has been a lack of identification and use of an internal control miRNA in bovine milk sEVs for normalization in a quantitative real-time polymerase chain reaction (qPCR).

qPCR is currently the most frequently used, highly sensitive, specific, reproducible, and accurate molecular technique used for the evaluation of miRNAs in various cells and tissues, as well as in bovine milk sEVs [11,12]. To remove non-biological variations, gene quantification with qPCR needs a precise normalizing procedure. Among the different ways now being addressed, the implementation of internal control genes, which should be expressed consistently in both the normal and pathological states of the host, is the suggested method of normalization [13,14,15]. Previous studies have reported that few miRNAs, such as miR-16, miR-423, miR-484, and non-coding RNAs such as U6, are often used as internal control miRNAs in plasma or serum [16]; however, these miRNAs may not be suitable for sEVs miRNA normalization in bovine milk due to the high complexities of bovine milk. Therefore, it is very difficult to identify reliable internal control miRNAs in bovine milk sEVs. This study aimed to identify an appropriate RNA extraction kit and suitable internal control miRNAs in bovine milk sEVs for normalization in a qPCR analysis. In this study, we compared two RNA extraction kits, the miRNeasy Micro Kit and Maxwell RSC miRNA Tissue Kit, in bovine milk sEVs. Five candidate internal control miRNAs in bovine milk sEVs were assessed by qPCR analysis, followed by the identification of their stability using geNorm [17], NormFinder [18], BestKeeper [19], and the ΔCt algorithm [20]. The present study indicated that the miRNeasy Micro Kit and Maxwell RSC miRNA Tissue Kit show better RNA recovery from bovine milk sEVs; however, the miRNeasy Micro Kit has higher efficiency among the two. A comprehensive stability ranking was finalized using the RefFinder software [21] which demonstrated that hsa-miR-27b and bta-miR-29a could be suitable internal control miRNAs using both kits. The study suggested that the use of the identified internal control miRNAs may improve the reliability and accuracy of the qPCR analysis for the normalization of miRNAs in bovine milk sEVs. Therefore, this report also contributes to helping bovine milk sEVs researchers in evaluating the decision-making protocols and validity of laboratory RNA extraction methods according to their requirements. This is the first investigation to uncover internal control miRNAs in bovine milk sEVs.

## 2. Materials and Methods

### 2.1. Clinical Status of the Cattle

#### 2.1.1. Blood Collection

Blood samples (10 mL) were collected from the coccygeal vein of 12 Holstein-Friesian dairy cattle, where six were uninfected and healthy and six were BLV-infected cattle. We used both healthy and BLV-infected cattle in our experiment to minimize experimental errors in qPCR analyses. Blood was directly transferred to vacuum blood collection tubes with or without an anticoagulant (heparin) (VP-AS076K, VP-NA050K, and VP-H070K, Terumo, Tokyo, Japan). White blood cells (WBCs) and lymphocyte counts were measured using an automatic cell counter (Celltac α; Nihon Kohden, Tokyo, Japan).

#### 2.1.2. Measurement of Lactate Dehydrogenase (LDH) Isozymes

After counting the WBCs and lymphocytes, 1.3 mL of each of the anticoagulated blood samples was centrifuged at 2500× *g* at 25 °C for 15 min for plasma separation by an MX-307 centrifuge (Tomy Seiko, Tokyo, Japan). The LDH isozymes in plasma from the uninfected and BLV-infected cattle were measured by a Hydrasys 2 Scan (Sebia, Paris, France) using HYDRAGEL 7 ISO-LDH (Sebia), conducted by a clinical laboratory testing company, Fujifilm Vet Systems (Tokyo, Japan).

#### 2.1.3. Identification of Anti-BLV Antibody in Serum

For serum separation, the coagulated blood was then centrifuged at 3000× *g* for 15 min at 25 °C using an MX-307 centrifuge. The levels of anti-BLV antibodies in the serum were measured using an anti-BLV antibody enzyme-linked immunosorbent assay (ELISA) kit (JNC, Tokyo, Japan) according to the manufacturer’s instructions.

#### 2.1.4. Extraction of DNA from WBCs

WBCs were isolated by hemolysis of the red blood cells with 0.83% ammonium chloride and 0.01% ethylenediaminetetraacetic acid (EDTA), followed by washing twice with a phosphate buffered saline (PBS). The total DNA was extracted from WBCs using a DNeasy Blood and Tissue Kit (69506; Qiagen, Hilden, Germany) according to the manufacturer’s instructions. The DNA concentration was measured using a NanoDrop Lite spectrophotometer (Thermo Fisher Scientific, Waltham, MA, USA).

#### 2.1.5. Detection of BLV Provirus and Measurement of BLV Proviral Load (PVL)

A nested polymerase chain reaction (nested PCR) was performed by amplification of the pX or envelope region of the BLV primer, according to a previously described protocol [22,23]. For the nested PCR, the reaction volume was 20 µL containing 0.5 U of polymerase from GoTaq Hot Start Green Master Mix (M5122, Promega, Madison, WI, USA), 0.5 µM each of forward and reverse primers, and 1 µL of extracted WBCs DNA (100 to 400 ng) using a thermal cycler (Applied Biosystems, Waltham, MA, USA). The thermal cycling conditions were as follows: 95 °C for 2 min, followed by 35 cycles of 94 °C for 45 s, 62 °C for 30 s, 72 °C for 30 s, and 72 °C for 4 min. The PVL of BLV-infected cattle was measured by a qPCR using a 5 µL template DNA sample. The reaction mixture contained 10 µL of THUNDERBIRD Probe qPCR Mix (A4250K, Toyobo, Osaka, Japan), 0.3 µL of CoCoMo-BLV Primer/Probe (A803, Riken Genesis, Tokyo, Japan), 5 µL of a template DNA sample, and PCR-grade water to increase the volume up to 20 µL. For the PVL quantification, BLV BoLA-DRA gene Plasmid DNA was obtained from the kit (A804, Riken Genesis) and the BLV proviral DNA was measured using a Thermal Cycler Dice Real Time System III (TP970, Takara Bio) according to the manufacturer’s instructions. The hematological tests, detection of serum antibodies against BLV, detection of BLV provirus, and measurement of PVL were conducted at the Gifu Chuo Livestock Hygiene Service Center (Gifu, Japan).

### 2.2. Milk Collection

Fresh raw milk was collected from 12 Holstein-Frisian cattle (six uninfected and six BLV-infected cattle) from different farms located in the Gifu Prefecture, Japan. At the time of milk collection, we found that the uninfected cattle no. 6 was in the early lactation stage, cattle nos. 1, 2, and 5 were in the mid-lactation stage, and cattle nos. 3 and 4 were in the late lactation stage. However, we did not have lactation stage data of the BLV-infected cattle. During and after milk collection from the cattle, all hygienic measurements were performed. The milk was collected from individual cattle by hand milking; hence, all milk samples were immediately transported to the laboratory in sterile jars and stored at 4 °C until further processing. All freshly collected bovine milk samples were pooled to eliminate variability in the milk composition across the cattle; however, they were processed separately to ensure independent sample extraction.

#### 2.2.1. Isolation and Characterization of Bovine Milk sEVs

Milk sEVs were isolated, purified, and characterized using the protocols described previously with slight modifications [24,25,26]. In brief, the milk fat was removed by using high speed centrifugation at 2000× *g* for 20 min by an MX-307 centrifuge (Tomy Seiko, Tokyo, Japan). After that, the upper fat was removed by a plastic disposable spoon and the defatted skim milk was pre-warmed at 37 °C for 10 min. For the efficient isolation of bovine milk sEVs, acetic acid was added (finally 1%) into the skim milk followed by the removal of casein using centrifugation at 5000× *g* for 20 min. The supernatant (whey) was collected and filtrated by using 1.0, 0.45, and 0.2 μm pore-size filters (GA-100, C045A047A, and C020A047A, Advantec, Tokyo, Japan). For the concentration of bovine milk sEVs, the whey was ultracentrifuged (UC) at 100,000× *g* at 4 °C for 1 h using a P42A angle rotor (Hitachi Koki, Tokyo, Japan) in a Himac CP80WX ultracentrifuge (Hitachi Koki). The supernatant solution was decanted and the bottom bovine milk sEVs pellet was collected. Later, distilled water (DW) was added to the bovine milk sEVs pellet up to 10 mL and recovered into a 13PET tube (Hitachi Koki). After that, UC was performed once again at 100,000× *g* at 4 °C for 1 h using a P40ST swing rotor (Hitachi Koki). The supernatant was aspirated by using a suction pump SP30 (Air Liquid Healthcare, Paris, France) and the bottom bovine milk sEVs pellet was collected for further use.

The bovine milk sEVs were further characterized by using transmission electron microscopy (TEM) and Western blotting (WB), as described previously [24,25,26] according to the Minimal Information for Studies of Extracellular Vesicles 2018 (MISEV2018) guidelines with slight modifications. The bovine milk sEVs pellet was diluted ten times with DW before being placed on glow-discharged carbon support films on copper grids. The bovine milk sEVs pellet solution was dyed with 2% uranyl acetate and placed in a silicon chamber for drying. Then, the bovine milk sEVs’ morphology was observed under an electron microscope, JEM-2100F (JEOL, Tokyo, Japan). In the WB analysis, after the electrophoresis and transfer of protein to polyvinylidene difluoride (PVDF) blotting membranes, the PVDF membranes were blocked for 20 min at room temperature (RT) with 5% skimmed milk. After that, the PVDF membranes were incubated with primary antibodies at RT for 1 h for the detection of a surface-marker-protein (anti-CD63, 1:200, M-13, SC-31214, Santa Cruz Biotechnology, Santa Cruz, CA, USA), internal-marker-protein (anti-HSP70, 1:100, N27F3-4, ADI-SPA-820, Enzo Life Science, Farmingdale, NY, USA), and contaminant protein (anti-apoA1, 1:100, B-10, SC-376818, Santa Cruz Biotechnology), respectively. The secondary antibodies (anti-goat IgG donkey antibody, 1:2000, SC-3851, Santa Cruz Biotechnology; anti-mouse IgG antibody, 1:2000, 7076, Cell Signaling Technologies, Danvers, MA, USA) conjugated with horseradish peroxidase were applied to the PVDF membrane. Labeled antibodies were detected using Pierce ECL Plus substrate (Thermo Fisher Scientific, Waltham, MA, USA) and the protein images were captured by a chemiluminescence imager, ChemiDoc XRS+ (Bio-Rad Laboratories, Hercules, CA, USA).

#### 2.2.2. Extraction of RNA from Bovine Milk sEVs

The total RNA was extracted from the bovine milk sEVs using the miRNeasy Micro Kit (217084, Qiagen) and Maxwell RSC miRNA Tissue Kit (AS1460, Promega), respectively, according to the manufacturer’s instructions. Immediately following extraction, the RNA concentration and A260/A280 ratio of each bovine milk sEVs sample were determined using a 2100 Agilent Bioanalyzer (Agilent Technologies, Santa Clara, CA, USA).

#### 2.2.3. Selection of Candidate Internal Control miRNAs in Bovine Milk sEVs

To select the candidate internal control miRNAs in bovine milk sEVs, previously published microarray raw data were compared and evaluated [6,7]. To identify the common miRNAs in the two datasets, the raw data of miRNAs were combined to generate a single dataset. miRNAs without a gene or dual name, but the same gene names, were not counted for analysis. All raw data were loaded into an Excel file, and the coefficient of variation (CV) value was calculated. Low CV values of the miRNAs were later selected for the identification of internal control miRNAs in bovine milk sEVs.

#### 2.2.4. Quantification of miRNAs in qPCR Analysis

The quantification of miRNAs in bovine milk sEVs was performed using the miRCURY LNA RT Kit (339340, Qiagen) and miRCURY LNA SYBR Green PCR Kit (339346, Qiagen). Briefly, the RNA concentration of all milk sEVs was adjusted to 20 ng/µL, subjected to reverse transcription in a 10 µL reaction solution, and incubated for 60 min at 42 °C, followed by further incubation at 95 °C for 5 min to inactivate the reverse transcriptase enzyme. Primers such as bta-miR-29a, bta-miR-200a, bta-miR-26b, bta-miR-27b, and bta-miR-30b-5p were contained in the miRCURY LNA miRNA PCR Assay components (339306, Qiagen). For the qPCR, 3 µL complementary DNA (cDNA) was added to the total volume of the reaction mixture. The qPCR was performed using a StepOnePlus analytical thermal cycler (Applied Biosystems, Foster City, CA, USA). The thermal cycling program was as follows: 95 °C for 2 min for initial heat activation, followed by 40 cycles of 95 °C for 10 s for denaturation, and 56 °C for 1 min for annealing and extension. The melting curve was analyzed at 95 °C for 15 s, 60 °C for 1 min, and 95 °C for 15 s. For each candidate internal control miRNA, the qPCR was performed in duplicates (technical replicates). The qPCR excel data for each candidate internal control miRNA were extracted and analyzed further.

#### 2.2.5. Assessment of Stability of Candidate Internal Control miRNAs

The stability of candidate internal control miRNAs in bovine milk sEVs were analyzed by using major computational programs currently available such as geNorm [17], NormFinder [18], BestKeeper [19], and the ΔCt [20] algorithms. Finally, a comprehensive stability ranking of suitable internal control miRNAs in bovine milk sEVs was analyzed using the RefFinder software [21] on the basis of the results obtained from the geNorm, NormFinder, BestKeeper, and ∆Ct algorithms.

## 3. Results

### 3.1. Clinical Status of the Cattle

The clinical statuses of the cattle are presented in Table 1. Nested PCR and ELISA confirmed the presence of a BLV infection in the cattle. The PVL results indicated that BLV-infected cattle 1 had a low PVL (BLV copies < 10,000/10^5^ WBCs) and other BLV-infected cattle had a high PVL (BLV copies ≥ 10,000/10^5^ WBCs) with a high LDH 2 + 3 percentage of over 30%.

### 3.2. Isolation and Characterization of Bovine Milk sEVs

**Figure 1:**
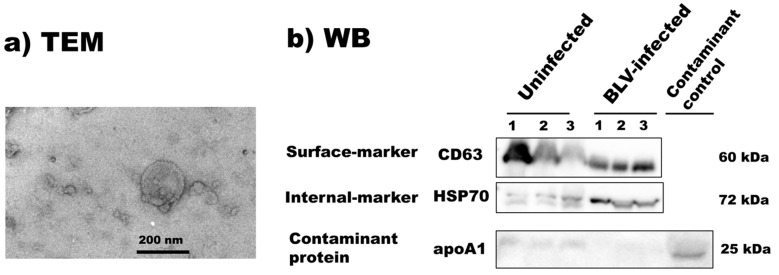
Characterization of bovine milk small extracellular vesicles. Morphology of bovine milk sEVs by transmission electron microscopy analysis (**a**) and detection marker proteins in bovine milk sEVs by Western blot analysis. (**b**) TEM detected bilayer spherical shape sEVs in milk (scale bar shows 200 nm). WB analysis successfully detected surface-marker-protein CD63 and internal-marker-protein HSP70 by using anti-CD63 and –HSP70 antibodies. WB analysis also detected less contaminant protein apoA1 in bovine milk sEVs. TEM, transmission electron microscopy; WB, Western blot.

From the transmission electron microscopy (TEM) analysis, the morphological features of bovine milk sEVs from uninfected and BLV-infected cattle presented in a similar spherical bilayer shape; a representative figure is shown in Figure 1 and Supplementary Material Figure S1. These results confirmed the presence of milk sEVs in the current study.

#### 3.2.1. Selection of Candidate Internal Control miRNAs in Bovine Milk sEVs

To identify the candidate internal control miRNAs in bovine milk sEVs, we compared the microarray raw data published previously [6,7]. A total of 786 miRNAs in bovine milk sEVs were identified as common between the two datasets (data are not shown). Furthermore, five miRNAs, bta-miR-29a, bta-miR-200a, bta-miR-26b, bta-miR-27b-3p, and bta-miR-30b-5p, were selected as the candidate internal control miRNAs in bovine milk sEVs based on their low CV values. Detailed primer information for candidate internal control miRNAs in bovine milk sEVs is shown in Table 2. Owing to the unavailability of bovine miRNAs, probes for human miRNAs were selected for their bovine counterparts.

#### 3.2.2. Comparison of RNA Extraction Kit

**Figure 2:**
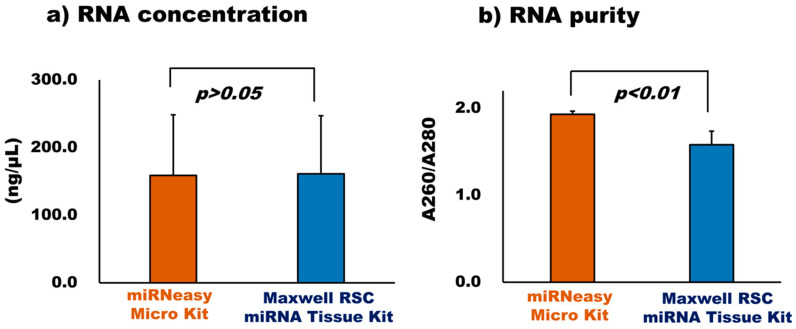
Comparison of RNA concentration (**a**) and RNA purity (**b**) in milk sEVs extracted by miRNeasy Micro Kit and Maxwell RSC miRNA Tissue Kit. Data are presented as a mean value for each kit. There was no significant difference in RNA concentration between the two kits. However, RNA purity was higher and significantly different in miRNeasy Micro Kit (A260/A280: 1.93) than that in Maxwell RSC miRNA Tissue Kit (A260/A280: 1.58) (*p* < 0.01).

**Figure 3:**
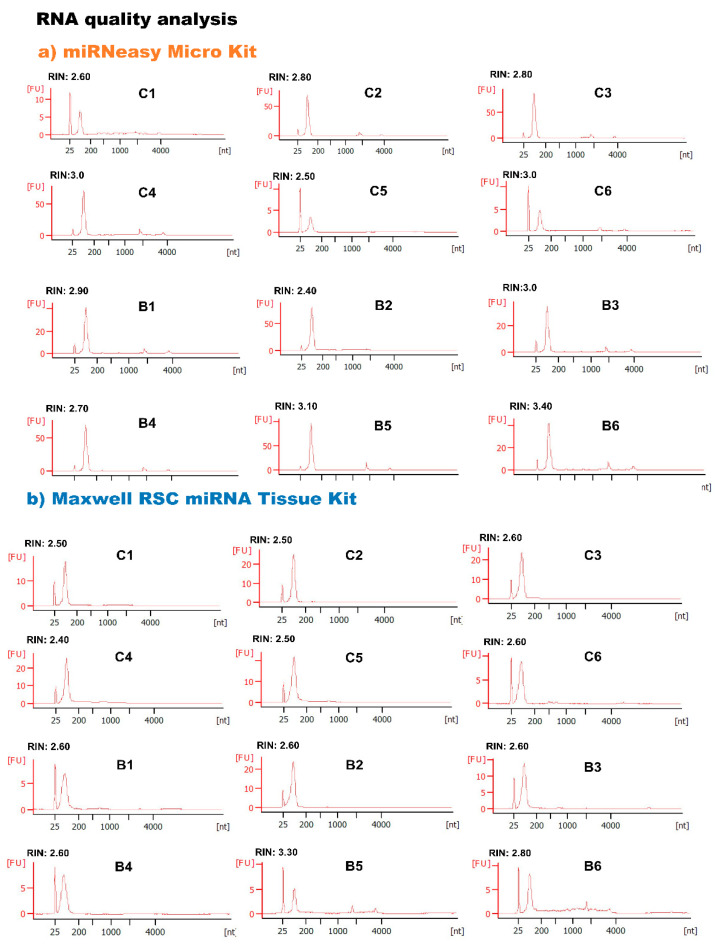
RNA quality analysis. The total RNA in bovine milk sEVs extracted by miRNeasy Micro Kit (**a**) and Maxwell RSC miRNA Tissue Kit (**b**) was analyzed for RNA quality assessment by using Total RNA Nano Kit (Agilent 2100 Bioanalyzer). The electropherograms demonstrated size distribution of nucleotides and fluorescence intensity value of total RNA in bovine milk sEVs. The peak at 25 nt indicated the internal standard. The 18s- and 28s-ribosomal RNA is a part of the mathematical algorithm where RIN values were calculated and being used as a measure of the RNA quality (RIN; 1 = totally degraded, 10 = intact). The RIN value from the extracted RNA was showing no 18s- and 28s-ribosomal RNA peaks. nt, nucleotides; FU, fluorescence intensity; RIN, RNA integrity number; c, uninfected control cattle; B, BLV-infected cattle.

**Figure 4:**
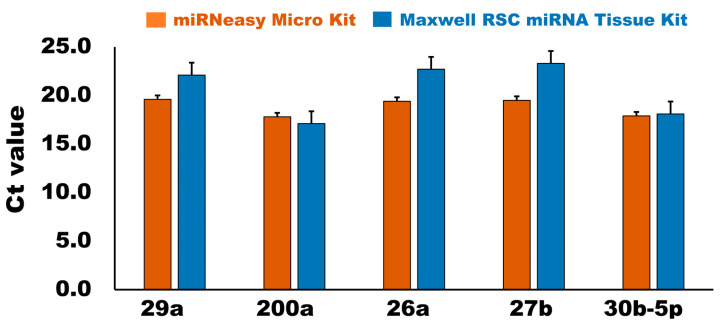
Comparison of cycle threshold (Ct) value of candidate internal control miRNAs in bovine milk sEV by qPCR analysis. In the case of the miRNeasy Micro Kit, average Ct value ranges from 17–20; whereas in the case of the Maxwell RSC miRNA Tissue kit, average Ct value ranges from 17–24, indicating that miRNeasy Micro Kit has better efficiency than that of Maxwell RSC miRNA Tissue Kit.

For the proper and efficient recovery of RNA from bovine milk sEVs, two different RNA extraction kits (miRNeasy Micro Kit and Maxwell RSC miRNA tissue kit) were compared (Figure 2, Figure 3 and Figure 4). The quantity of the RNA extracted using the two kits was assessed using the Bioanalyzer 2100. The RNA concentrations of the two kits were almost similar (*p* > 0.05). However, the A260/A280 ratio in the miRNeasy Micro Kit (A260/A280:1.93) was higher than that in the Maxwell RSC miRNA Tissue Kit (A260/A280:1.58), and their results were significantly different (*p* < 0.01). The results indicate that both RNA extraction kits have the potential to recover a greater amount of RNA from bovine milk sEVs. However, a higher RNA purity was obtained using the miRNeasy Micro Kit, indicating its better efficiency for RNA extraction from bovine milk sEVs. Total RNA was extracted from the bovine milk.

sEVs using the miRNeasy Micro Kit and the Maxwell RSC miRNA Tissue Kit, and RNA quality were assessed using a Bioanalyzer with RNA (RIN; 1 = completely degraded, 10 = intact). This algorithm is dependent on ribosomal RNA, while bovine milk sEVs lack ribosomal RNA (Figure 3). The results indicated that the extracted RNA in the bovine milk sEVs is of good quality.

#### 3.2.3. qPCR Analysis

Based on the microarray data of the bovine milk sEVs obtained from our previous studies [6,7], five candidate internal control miRNAs in bovine milk sEVs (*n* = 12) were quantified by the qPCR. All candidate internal control miRNAs were detected in the bovine milk sEVs using a qPCR. The cycle threshold (Ct) values of the candidate internal control miRNAs in the bovine milk sEVs are shown in Figure 4. All candidate internal control miRNAs had Ct values in the qPCR analysis. The range of Ct values indicated the stability of the candidate internal control miRNA in the qPCR. As the range of Ct values became wider for the miRNAs, the more unstable the miRNAs were during the qPCR. The Ct value ranged from 16.80 to 20.94 and 13.52 to 29.23 in the case of the miRNeasy Micro Kit and Maxwell RSC miRNA Tissue Kit, respectively. The results indicate that the Ct value of all the candidate internal controls was low and consistent in the miRNeasy Micro Kit compared with that of the Maxwell RSC miRNA Tissue Kit in the qPCR analysis. The most highly expressed miRNAs were 200a and 30b-5p, which had average Ct values of 17.8 and 17.1 in the case of the miRNeasy Micro Kit and 17.1 and 18.1 in the case of the Maxwell RSC miRNA Tissue Kit, respectively. A calculation of the Ct value is important for selecting an internal control standard. However, simple and comprehensive raw data of the Ct value for the identification of the candidate internal control miRNAs is insufficient. Therefore, we conducted an additional analysis using four different statistical algorithms to assess the candidate internal control miRNAs in bovine milk sEVs.

#### 3.2.4. Evaluation of Stability of the Candidate Internal Control miRNAs

There were four different distinct statistical algorithms, geNorm [17], NormFinder [18], BestKeeper [19], and the ΔCt algorithm [20], used to assess the suitability and stability of the candidate internal control miRNAs in bovine milk sEVs.

##### GeNorm Analysis

**Figure 5:**
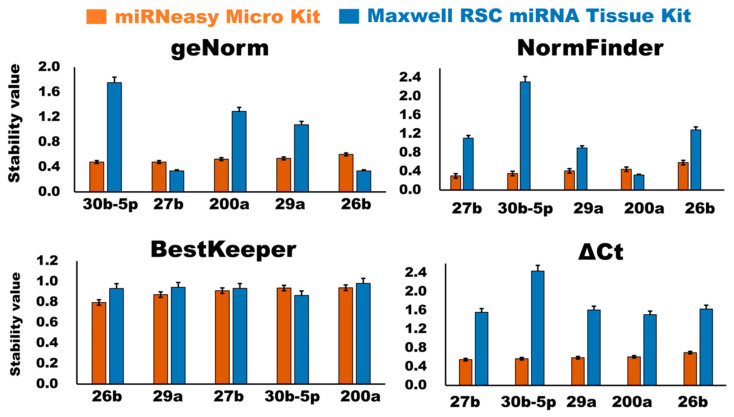
Stability analysis of candidate internal control miRNAs in bovine milk sEVs in accordance with extraction kits, miRNeasy Micro Kit and Maxwell RSC miRNA Tissue Kit by geNorm, NormFinder, BestKeeper, and ∆Ct. The *x*- and *y*-axis indicate the candidate internal control miRNAs and stability value of the miRNAs, respectively.

The geNorm algorithm [17] was used to evaluate the stability of the candidate internal control miRNAs by generating stability values (M-values). The higher the M-value, the lower the stability, and vice versa. The results of the geNorm analysis revealed that in the case of the miRNeasy Micro Kit, the stability ranking of the candidate internal control miRNAs in the bovine milk sEVs was 30b-5p, 27b, 200a, 29a, and 26b (Figure 5). In the case of the Maxwell RSC miRNA Tissue kit, the stability rankings of the candidate internal control miRNAs were 26b, 27b, 29a, 200a, and 30b-5p. The results indicated that the top three candidate internal control miRNAs from both kits would be suitable for normalization in the qPCR.

##### NormFinder Analysis

The NormFinder algorithm [18] was used to access and evaluate the stability of the candidate internal control miRNAs in milk sEVs (Figure 5). This program ranked candidate internal control miRNAs based on their grouping variations, with a lower stability value indicating high stable miRNAs in bovine milk sEVs. NormFinder ranked the five candidate internal control miRNAs in bovine milk sEVs, from the lowest to highest stability value in the case of the miRNeasy Micro Kit, as follows: 27b, 30b-5p, 29a, 200a, and 26b. In the case of the Maxwell RSC miRNA Tissue kit, the stability rankings of the candidate internal control miRNAs were 200a, 29a, 27b, 26b, and 30b-5p. The results indicated that the top three candidate internal control miRNAs from both kits would be suitable for normalization in the qPCR.

##### BestKeeper Analysis

The BestKeeper algorithm [19] analyzed the gene expression variation for the candidate internal control miRNAs in bovine milk sEVs by calculating the standard deviation (SD) and pairwise correlation (r) values. The lowest SD value indicated highly stable candidate internal control miRNAs, and vice versa. BestKeeper identified five candidate internal control miRNAs in the bovine milk sEVs (Figure 5). For the miRNeasy Micro Kit, the ranking of the candidate internal control miRNAs in bovine milk sEVs was 26b, 29a, 27b, 30b-5p, and 200a. In the case of the Maxwell RSC miRNA Tissue kit, the stability rankings of the candidate internal control miRNAs were 30b-5p, 26b, 27b, 29a, and 200a.

##### ΔCt Analysis

In the ΔCt algorithm [20], the mean SD was utilized to evaluate the stability of the candidate internal control miRNAs. A lower SD value indicated higher stable internal control miRNAs and vice versa.

The stability value of the internal control miRNAs according to the ΔCt algorithm is demonstrated in Figure 5. For the miRNeasy Micro Kit, the rankings of the candidate internal control miRNAs in the bovine milk sEVs were: 27b, 30b-5p, 29a, 200a, and 26b. In the case of the Maxwell RSC miRNA Tissue kit, the stability rankings of the candidate internal control miRNAs were 200a, 27b, 29a, 26b, and 30b-5p.

##### RefFinder Analysis

The use of various algorithms, such as geNorm, NormFinder, BestKeeper, and ΔCt, were revealed in the different candidate internal control miRNAs in bovine milk sEVs. Therefore, the comprehensive ranking of the candidate internal control miRNAs in bovine milk sEVs was determined using the RefFinder software [21]. The overall final rankings are listed in Table 3. The comprehensive rankings of the five candidate internal control miRNAs in bovine milk sEVs in the case of the miRNeasy Micro Kit (Table 3) were: 27b > 30b-5p > 29a > 200a > 26b, and in the case of the Maxwell RSC miRNA Tissue kit (Table 3) were: 200a > 27b > 29a > 26b > 30b-5p.

## 4. Discussion

In this study, we attempted to compare two commercially available miRNA extraction kits, such as the miRNeasy Micro Kit and Maxwell RSC miRNA Tissue Kit. Hence, five candidate internal control miRNAs were selected from previously reported microarray raw data [6,7]. Therefore, we compared two RNA extraction kits in the qPCR analysis for the identification of stable internal control miRNAs in bovine milk sEVs. The study indicated that the Maxwell RSC miRNA Tissue Kit showed a relatively higher RNA concentration than the miRNeasy Micro Kit (*p* > 0.05). However, the A260/A280 ratios were significantly higher in the miRNeasy Micro Kit than in the Maxwell RSC miRNA Tissue Kit (*p* < 0.01). The miRNeasy Micro Kit uses a phenol and pure column-based technique, whereas the Maxwell RSC miRNA Tissue Kit uses an automated paramagnetic technique. Previous studies reported that the miRNeasy kit yielded relatively high RNA concentrations with a high purity which is in accordance with the results of this study [28,29,30,31]. The present study identified that RNA purity was comparably low using the Maxwell RSC miRNA Tissue Kit. However, a previous study mentioned the usefulness of this kit based on safety associated with not handling chloroform and phenol [32]. Further, the quality of the extracted RNA was analyzed by Bioanalyzer and found no cellular RNA peaks in the bovine milk sEVs. These results were consistent with the previously published report [33]. The study reported that phenol-based and combined phenol and column based RNA extraction method efficiently extracted RNA from the sEVs that were lacking cellular RNA content, making it a useful tool for RNA extraction. Therefore, it is postulated that there may be a high variability in RNA concentration due to differences in the species, sample, and state of the host, which influences the kit’s performance and its success [33,34,35].

In this study, we investigated the suitability of five candidate internal control miRNAs: 29a, 200a, 26b, 27b, and 30b-5p, for the normalization of bovine milk sEVs by a qPCR. Therefore, this study employed multiple algorithms, such as geNorm [17], NormFinder [18], BestKeeper [19], and the ΔCt [20] algorithm, to identify the most suitable internal control miRNAs in bovine milk sEVs after obtaining the Ct values in a qPCR analysis. geNorm and BestKeeper are pair-wise correlation-based, and select the most appropriate internal control miRNAs for the variation of expression ratios among genes throughout the different sets of samples, although geNorm comprises pairings of co-regulated genes based on their similar expression pattern. NormFinder and the comparative ∆Ct method were used to eliminate the effects of co-regulation. The geNorm algorithm demonstrated that the stability rankings of the candidate internal control miRNAs in bovine milk sEVs were 30b, 27b, and 200a while using the miRNeasy Micro Kit and 26b, 27b, and 29a using the Maxwell RSC miRNA Tissue Kit, respectively. In geNorm, the best combination of two internal control miRNAs was 30b and 27b for the miRNeasy Micro Kit and 26b and 27b for the Maxwell RSC miRNA Tissue Kit. Both the geNorm and NormFinder algorithms recommended a cut-off value of 0.15 for the stability value [17,18] and all internal control miRNAs from the present study exceeded the cut-off value. In the geNorm algorithm, the most significant criterion for assessing the internal control miRNAs is the use of a pair-wise comparison strategy for obtaining the gene stability values [17]. On the other hand, NormFinder uses internal control miRNAs using an intra- and inter-group variance technique, eliminating the impact of gene co-regulation [18]. In this technique, appropriate internal control miRNAs were predicted to have stable values, as demonstrated by the low variation in the sample. The computation using NormFinder algorithms [18] revealed that all five selected miRNAs had a stability value of less than 1.0 in case of the miRNeasy Micro Kit, indicating that these miRNAs can be used as an internal control miRNA. However, in the case of the Maxwell RSC miRNA Tissue Kit, NormFinder revealed that only two candidate internal control miRNAs, 200a and 29a, had a stability value of less than 1.0, indicating that these two can be used as internal control miRNAs in milk sEVs. Similarly, the qPCR analysis also proved that all candidate internal control miRNAs had low and consistent Ct values in the miRNeasy Micro Kit compared to the Maxwell RSC miRNA Tissue Kit.

According to the BestKeeper algorithm, 26b, 29a, and 27b were identified as highly stable internal control miRNAs in the bovine milk sEVs using the miRNeasy Micro Kit, and 30b-5p, 26b, and 27b were identified as highly stable internal control miRNAs in the bovine milk sEVs using the Maxwell RSC miRNA Tissue Kit. In BestKeeper, appropriate internal control miRNAs are anticipated to have adequate stable values, as shown by the minimum variation in the samples under assessment [19]. Furthermore, in BestKeeper, Pearson correlation coefficients (r) and *p*-values are the two most significant criteria for assessing the stability of the internal control miRNAs. This algorithm performs a pair-wise correlation analysis for all possible pairs of candidate internal control miRNAs according to the raw Ct values and generates the geometric mean of the finest ones. In the ∆Ct method, the candidates for highly stable internal control miRNAs in bovine milk sEVs were 27b, 30b-5p, and 29a in the case of the miRNeasy Micro Kit and 200a, 27b, and 29a in the case of the Maxwell RSC miRNA Tissue Kit. In this algorithm, the calculations were carried out based on the internal control miRNAs providing raw Ct values in the qPCR [20]. Unfortunately, we were not able to find any internal control miRNAs that were generally constant and stable in all four algorithms. This is because different algorithms use different computations and approaches based on a pair-wise or model-based comparison, leading to inconsistent results. To overcome this constraint, the RefFinder software [21] was used to determine the ranking of stable internal control miRNAs in bovine milk sEVs. The findings indicated that 27b, 30b-5p, and 29a are highly stable internal control miRNAs in bovine milk sEVs in the case of the miRNeasy Micro Kit and 200a, 27b, and 29a in the case of the Maxwell RSC miRNA Tissue Kit, respectively, for normalizing miRNA for a qPCR.

We did not compare our identified results with those of previous studies due to the lack of published reports. This study identified candidate internal control miRNAs which have not been previously reported as internal control miRNAs, as normalizer genes in a qPCR. However, studies have reported that the candidate internal control miRNAs identified in this study, such as miR-29a, miR-200a, miR-26b, miR-27b-3p, and miR-30b-5p, are dysregulated in many cancer tissues, cancer cell lines, and metastasis [36,37,38,39]. Up until now, a large number of studies have been published in an attempt to uncover tissue- and cell-specific or universal reference genes; however, difficulty persists. Various controversies have been reported previously. For example, miR-16 is frequently used as an internal control miRNA in cancer research; however, another study indicated that miR-16 has potential roles in controlling the cell cycle, promoting apoptosis, and reducing tumorigenicity [40]. A previous study showed that miR-16 can be useful for biomarker potentiality across a variety of cancer types, while another study used the same miRNA as an internal control gene in breast cancer for normalization in a qPCR [41]. Studies reported that miRNA-191 is widely used as an internal control gene in various cancers, including ovarian and cervical cancers [42,43]. However, earlier studies have linked miRNA-191 to a variety of diseases [44]. A non-coding RNA such as U6 has previously been reported to be used as an internal control gene [45]. Given the fact that non-coding RNAs are not in the same class of RNA as miRNAs, their compositions and properties are not the same, which may lead to misleading results during qPCR analysis. A different explanation could be related to variations in the methods used to quantify miRNAs in qPCR analysis. Even if the samples are collected from the same host but different tissue or cell types, heterogeneity may arise due to the pathophysiological status [40,41]. As a result, this study identified candidate internal control miRNAs that could be useful as moralize genes in bovine milk sEVs for qPCR analysis. However, the identified internal control miRNAs should not be useful as internal control miRNAs other than bovine milk sEVs. The current study has a few limitations. A small number of bovine milk sEVs samples were used, compared to only two RNA extraction kits, and the number of candidate internal control miRNAs was limited to five. Hence, extensive assessments of the candidate internal control miRNAs should be performed according to a specific experimental procedure and layout using large-scale experimental samples by qPCR analysis.

## 5. Conclusions

In conclusion, this study indicates that the miRNeasy Micro Kit and Maxwell RSC miRNA Tissue Kit show efficient RNA recovery from bovine milk sEVs; however, the miRNeasy Micro Kit is better for RNA extraction from bovine milk sEVs. It also suggests that due to the stability ranking value in both kits, hsa-miR-27b and bta-miR-29a could be utilized as internal control miRNAs in bovine milk sEVs for normalization in a qPCR. Further research is required to validate the candidate potency of these miRNAs as internal control miRNAs in bovine milk sEVs. To the best of our knowledge, this is the first investigation to discover suitable internal control miRNAs for bovine milk sEVs.

## Figures and Tables

**Table 1:** Clinical status of the cattle in this study.

Cattle	Age (Month)	ELISA	Nested PCR	PVL (Copies/10^5^ WBC)	WBC (/µL)	Lymphocyte (/µL)	Key of EC	Total LDH (U/L)	LDH Isozyme (%)					
									1	2	3	2 + 3	4	5
	**Uninfected cattle**													
1	19	−	−	−	4800	2700	−	881	51.5	26.4	14.9	41.3	4.9	2.3
2	20	−	−	−	9100	4400	−	955	50.0	25.9	15.4	41.3	5.2	3.5
3	31	−	−	−	8600	4200	−	876	52.4	22.6	15.7	38.3	5.2	4.1
4	66	−	−	−	6000	3100	−	839	58.8	25.1	12.4	37.5	2.8	0.9
5	42	−	−	−	5400	2100	−	778	57.5	25.6	12.6	38.2	2.9	1.4
6	115	−	−	−	NT	NT	−	723	63.9	16.6	13.0	29.6	4.3	2.2
	**BLV-infected cattle**													
1	52	+	+	9057.53	7400	3656	−	882	55.9	22.0	13.6	35.6	5.0	3.5
2	22	+	+	21,589.79	12,300	6950	−	917	53.3	29.7	13.3	43.0	3.1	0.6
3	55	+	+	27,029.76	18,800	10,171	+	1124	60.7	22.9	9.7	32.6	2.6	4.1
4	41	+	+	31,802.33	10,600	7378	±	1129	52.5	21.7	15.3	37.0	6.4	4.1
5	52	+	+	47,450.30	11,000	6666	±	1221	60.3	22.2	11.0	33.2	4.4	2.1
6	53	+	+	87,417.31	24,400	13,713	+	1233	58.1	22.0	13.0	35.0	4.6	2.3

+ positive; − negative; NT, not tested; BLV, bovine leukemia virus; sEVs, small extracellular vesicles; qPCR, quantitative real-time polymerase chain reaction; ELISA, enzyme-linked immunosorbent assay; PVL, proviral load; WBCs, white blood cells; Key of EC, leukosis-key of the European Community (−, normal; ±, suspect) [27]; LDH, lactate dehydrogenase.

**Table 2:** Primer information of internal control miRNA candidates in bovine milk sEVs.

miRNAs Name	Symbolic Presentation	Primers (Qiagen)	GeneGlobe ID
bta-miR-29a	29a	bta-miR-29a miRCURY LNA miRNA PCR Assay	YP02114732
bta-miR-200a	200a	bta-miR-200a miRCURY LNA miRNA PCR Assay	YP02104134
bta-miR-26b	26b	bta-miR-26b miRCURY LNA miRNA PCR Assay	YP00205953
bta-miR-27b	27b	hsa-miR-27b-3p miRCURY LNA miRNA PCR Assay	YP00205915
bta-miR-30b-5p	30b-5p	hsa-miR-30b-5p miRCURY LNA miRNA PCR Assay	YP00204765

bta, *Bos taurus*; hsa, *Homo sapiens*.

**Table 3:** Internal control miRNA candidates in bovine milk sEVs stability ranking by RefFinder analysis.

	Stability Ranking				
Name of RNA extraction kit	1	2	3	4	5
miRNeasy Micro Kit	miR-27b-3p	miR-30b-5p	miR-29a	miR-200a	miR-26b
Maxwell RSC miRNA Tissue kit	miR-200a	miR-27b-3p	miR-29a	miR-26b	miR-30b-5p

## Data Availability

The data presented in this study are available within the article and in the Supplementary Material.

## Supplementary Materials

The following supporting information can be downloaded at: https://www.mdpi.com/article/10.3390/membranes13020185/s1, Supplementary Material Figure S1. Full length Western blot images of bovine milk sEVs—surface-marker-protein CD63, internal-marker-protein HSP70 and contaminant control-marker-protein apoA1 were demonstrated. The WB images were taken by a chemiluminescence imager, ChemiDoc XRS+ (Bio-Rad Laboratories) and the molecular weight of the marker band was highlighted by using a LuminolPen (LH03-50, Visual Protein Biotechnology Corp., Taipei, Taiwan). WB, Western blot.
