# Supplementary figures and images for: Identification of Suitable Internal Control miRNAs in Bovine Milk Small Extracellular Vesicles for Normalization in Quantitative Real-Time Polymerase Chain Reaction
Source: Membranes (Basel). 2023 Feb 2;13(2):185. doi: 10.3390/membranes13020185 (PMC9961204; doi:10.3390/membranes13020185)

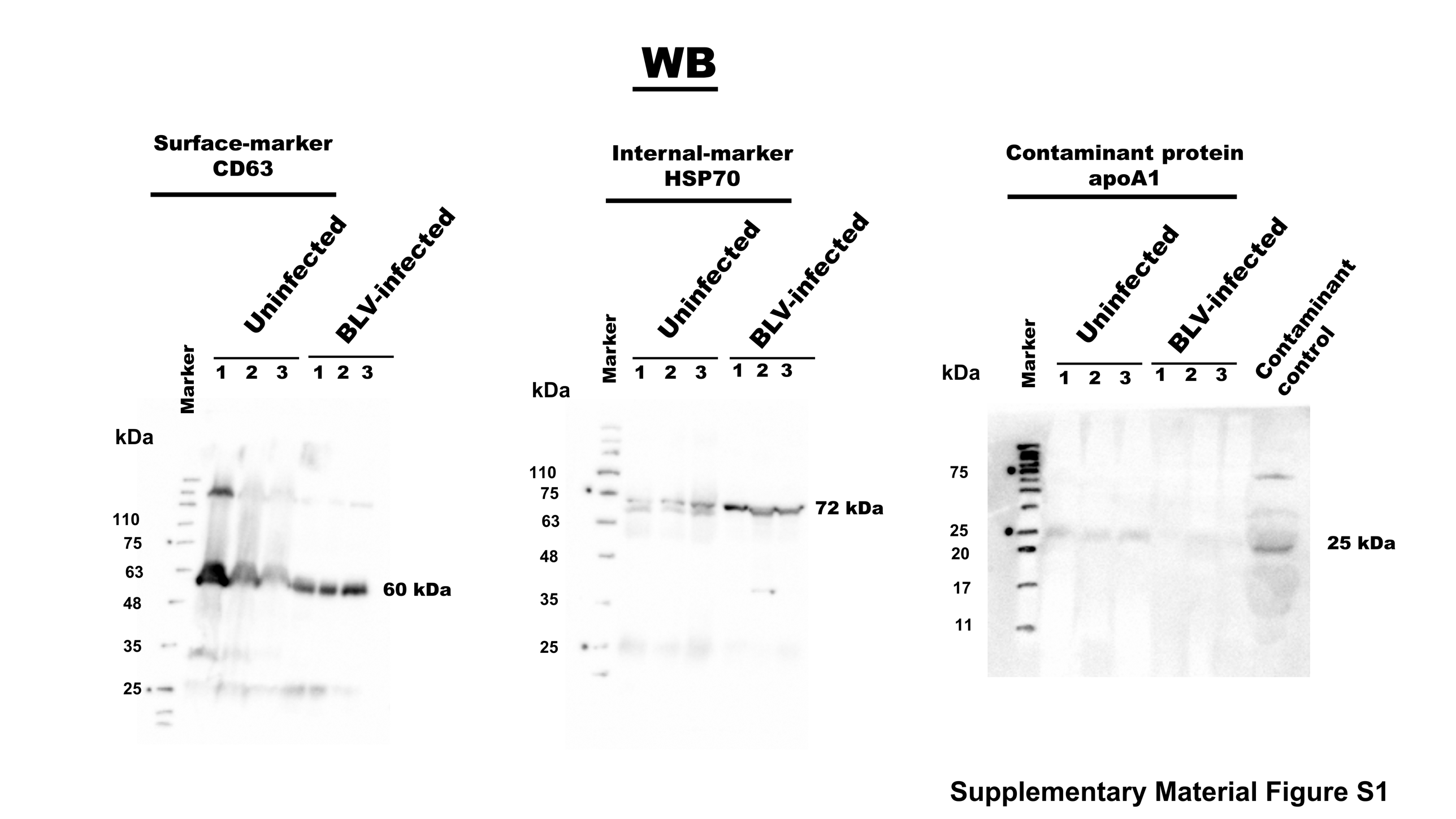

Supplement: Supplementary file 1 [file membranes-13-00185-s001.zip › membranes-2097797-supplementary.png]
